# Supplementary material for: Flagellar cAMP signaling controls trypanosome progression through host tissues
Source: Nat Commun. 2019 Feb 18;10:803. doi: 10.1038/s41467-019-08696-y (PMC6379439; doi:10.1038/s41467-019-08696-y)
Supplement: Supplementary file 1 — Supplementary Information [file 41467_2019_8696_MOESM1_ESM.pdf]

Flagellar cAMP signaling controls trypanosome progression through host tissues

Shaw and DeMarco et al.

Supplementary Figure 1

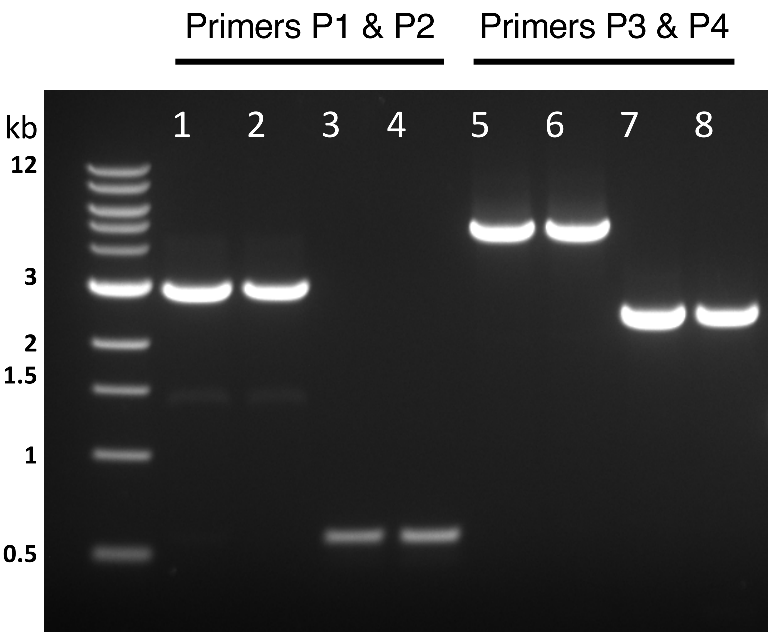

1. WT
2. WT-dsRed
3. PDEB1 KO
4. PDEB1 KO-dsRed
5. WT
6. WT-dsRed
7. PDEB1 KO
8. PDEB1 KO-dsRed

Primer 1: PDEB1 ORF fwd
Primer 2: PDEB1 ORF rev

Primer 3: upstream of 5' homologous region in plasmid
Primer 4: downstream of 3' homologous region in plasmid

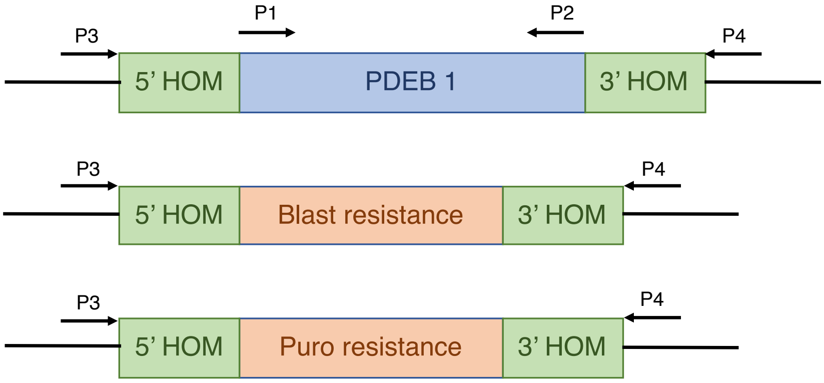

PCR confirmation of PDEB1 knockout.
Genomic DNA was isolated from individual clones and amplified with the primers shown above.

Supplementary Figure 2

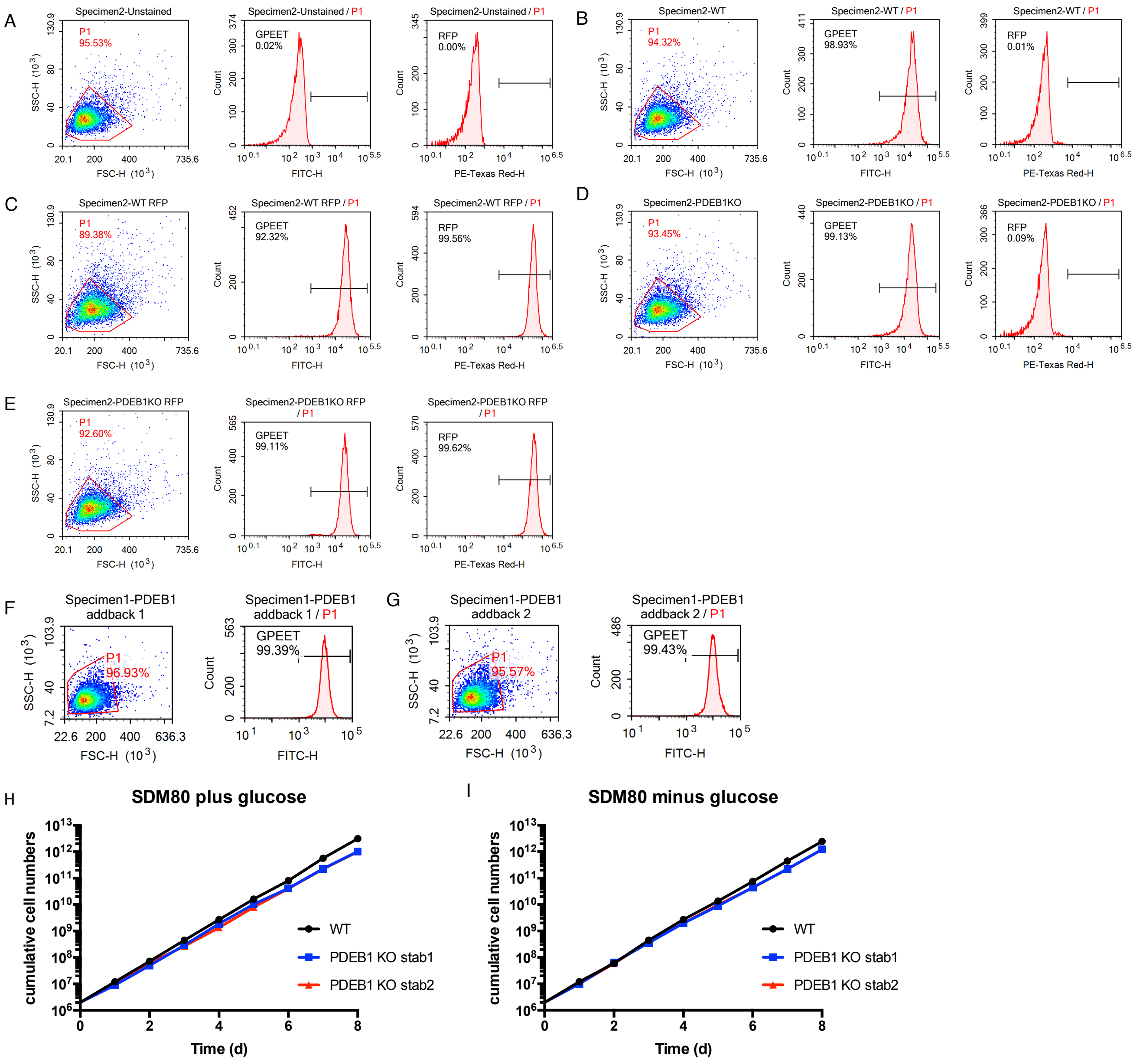

Analysis of expression of GPEET and dsRed by flow cytometry of all clones used in this study and growth of wild type and PDEB1 knockout in low glucose medium.

Left panels: gated population (indicated in red) used for analysis. Middle panels: GPEET surface staining. Right panels: dsRed expression. A) untagged WT (no antibody control). B) untagged WT. C) WT-dsRed. D) untagged PDEB1 KO. E) PDEB1 KO-dsRed. F) Addback 1. G) Addback 2.

Comparison of population doubling times of wild-type cells and two stabilates of PDEB1 KO either with glucose (H) or without glucose (I).

Supplementary Figure 3

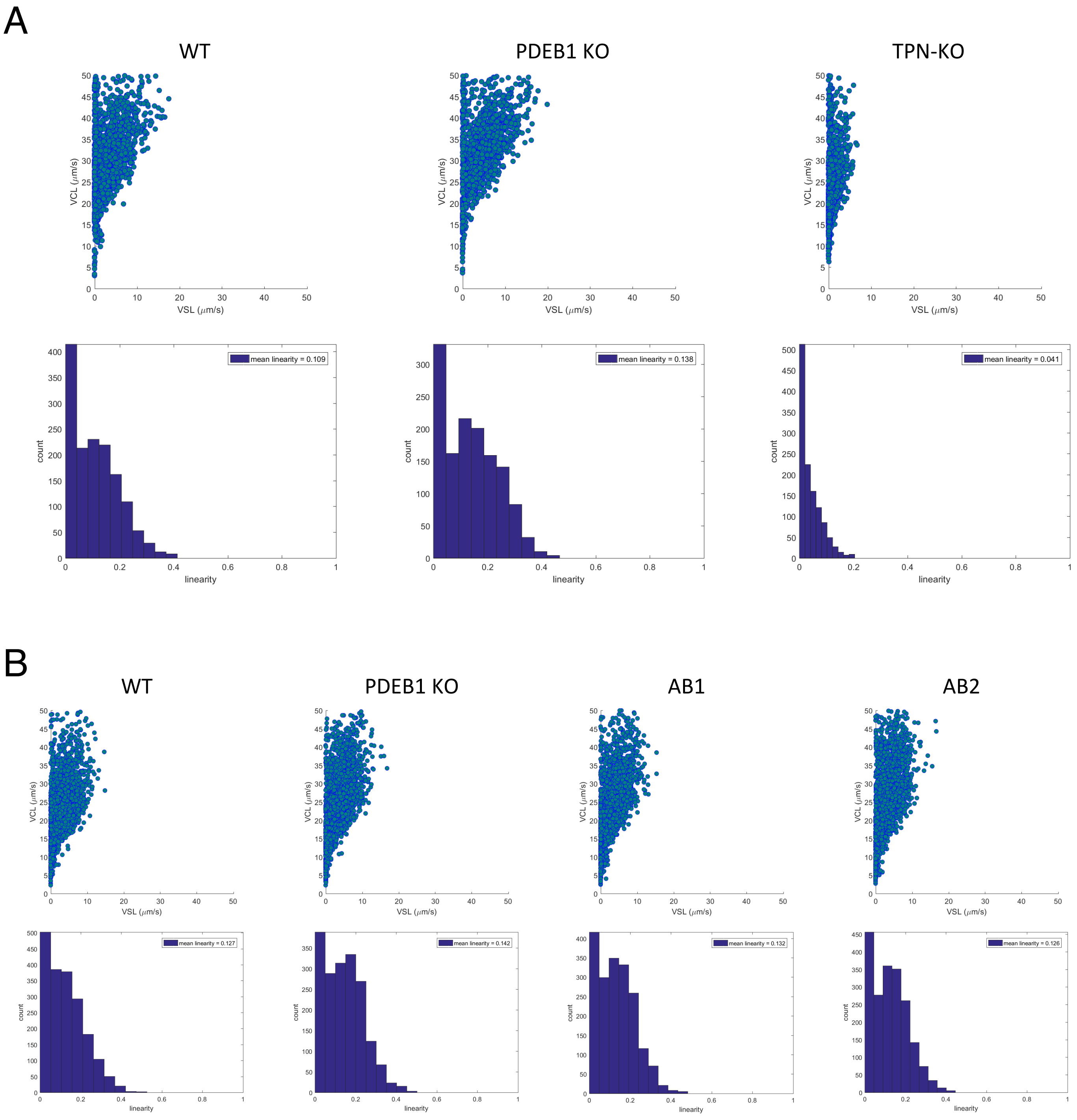

PDEB1 KO cells move more linearly than WT which contributes to their increased MSD
(A) Upper row: The curvilinear velocity (VCL) and the straight-line velocity (VSL) for each individual cell are plotted for WT (n=1449), PDEB1 KO (n=1339), and TPN KO (n=1208) respectively. Each blue dot corresponds to one cell. Lower row: Linearity plots for WT, PDEB1 KO, and TPN KO show how linear the motion of the cells in each population (WT, PDEB1 KO, or TPN KO) are. Mean linearity is the ratio of VSL/VCL for the cells in the population.
(B) Upper row: VCL and VSL are plotted for WT (n=1919), PDEB1 KO (n=1824), AB1 (n=1876), and AB2 (n=1973). Lower row: Linearity plots for WT, PDEB1 KO, AB1, and AB2.

Supplementary Figure 4

A

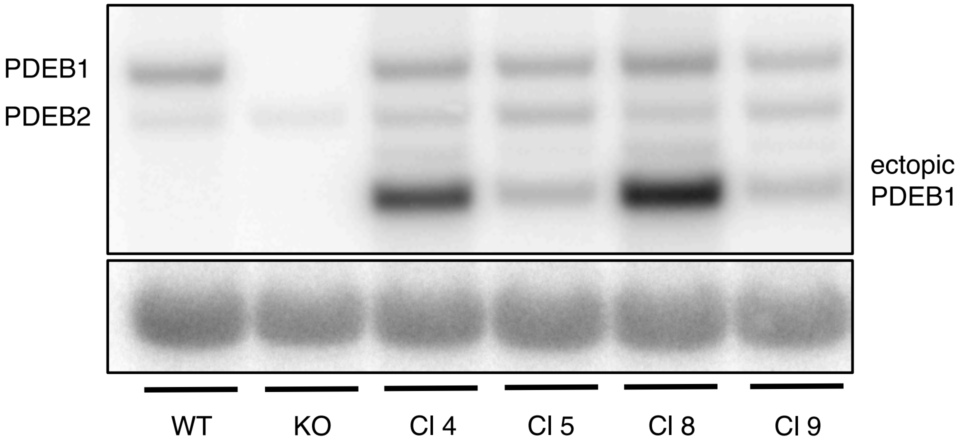

B

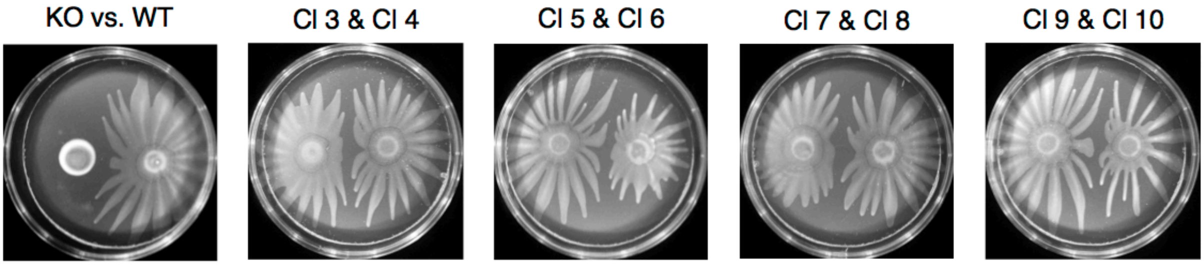

Overexpression of PDEB1 in WT cells has no adverse effects on social motility
(A) Northern blot analysis with a probe specific for the PDEB1 open reading frame (upper panel) and a loading control (18S rRNA, lower panel). WT: wild-type parent, KO: knockout. Cl4, Cl5, Cl8, and Cl9 are derivatives of WT stably transformed with the same plasmid that was used to generate AB1 and AB2 (see Figure 4). Note that this northern blot and the one shown in Figure 4A were part of the same gel and contain the same controls. (B) Representative pictures of social motility assays of KO, WT, and 8 overexpressors (Cl3-Cl10).

Supplementary Figure 5

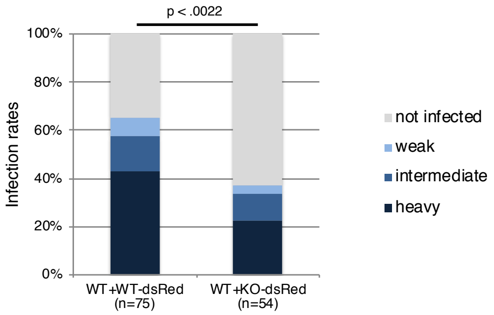

Total midgut infection rates for mixed infections with untagged WT and dsRed-tagged WT or KO (see Results, section 5). Fisher's exact test, two-sided was applied.
