# Supplementary material for: Flagellar cAMP signaling controls trypanosome progression through host tissues
Source: Nat Commun. 2019 Feb 18;10:803. doi: 10.1038/s41467-019-08696-y (PMC6379439; doi:10.1038/s41467-019-08696-y)
Supplement: Supplementary file 3 — Description of Additional Supplementary Files [file 41467_2019_8696_MOESM3_ESM.pdf]

Description of Additional Supplementary Files

File Name: Supplementary Movie 1

Description: Compilations of high-speed videos of WT, PDEB1 KO, and TPN KO cells. Videos were recorded at 496 frames per second (fps) and played back at 30 fps. Movie 1 includes four representative videos of WT cells, Movie 2 shows five representative videos of PDEB1 KO, and Movie 3 has five representative videos of TPN KO

File Name: Supplementary Movie 2

Description: Compilations of high-speed videos of WT, PDEB1 KO, and TPN KO cells. Videos were recorded at 496 frames per second (fps) and played back at 30 fps. Movie 1 includes four representative videos of WT cells, Movie 2 shows five representative videos of PDEB1 KO, and Movie 3 has five representative videos of TPN KO

File Name: Supplementary Movie 3

Description: Compilations of high-speed videos of WT, PDEB1 KO, and TPN KO cells. Videos were recorded at 496 frames per second (fps) and played back at 30 fps. Movie 1 includes four representative videos of WT cells, Movie 2 shows five representative videos of PDEB1 KO, and Movie 3 has five representative videos of TPN KO

File Name: Supplementary Movie 4

Description: Proventriculus of a fly co-infected with untagged WT and PDEB1 KO-dsRed at day 14 post infection. Scale bar: 100 microns

File Name: Supplementary Movie 5

Description: Z-stacks of proventriculi from flies infected with WT-dsRed at day 14 post infection. Scale bar: 100 microns

File Name: Supplementary Movie 6

Description: Z-stacks of proventriculi from flies infected with WT-dsRed at day 14 post infection. Scale bar: 100 microns

File Name: Supplementary Movie 7

Description: Z-stacks of midguts infected with WT-dsRed at day 14 post infection. The peritrophic matrix is stained with FITC-WGA and the nuclei of midgut endothelial cells are stained with Hoechst dye. Scale bar: 100 microns. Movie 7: Arrow indicates parasites in the ectoperitrophic space (EPS). Movie 8: 1st arrow indicates parasites in the midgut, and 2nd arrow points to parasites in the EPS. Movie 9: 1st arrow shows parasites in the EPS, and the 2nd and 3rd arrows point to parasites in the midgut

File Name: Supplementary Movie 8

Description: Z-stacks of midguts infected with WT-dsRed at day 14 post infection. The peritrophic matrix is stained with FITC-WGA and the nuclei of midgut endothelial cells are stained with Hoechst dye. Scale bar: 100 microns. Movie 7: Arrow indicates parasites in the ectoperitrophic space (EPS).

Movie 8: 1st arrow indicates parasites in the midgut, and 2nd arrow points to parasites in the EPS. Movie 9: 1st arrow shows parasites in the EPS, and the 2nd and 3rd arrows point to parasites in the midgut

File Name: Supplementary Movie 9

Description: Z-stacks of midguts infected with WT-dsRed at day 14 post infection. The peritrophic matrix is stained with FITC-WGA and the nuclei of midgut endothelial cells are stained with Hoechst dye. Scale bar: 100 microns. Movie 7: Arrow indicates parasites in the ectoperitrophic space (EPS). Movie 8: 1st arrow indicates parasites in the midgut, and 2nd arrow points to parasites in the EPS. Movie 9: 1st arrow shows parasites in the EPS, and the 2nd and 3rd arrows point to parasites in the midgut
