# Supplementary material for: Flagellar cAMP signaling controls trypanosome progression through host tissues
Source: Nat Commun. 2019 Feb 18;10:803. doi: 10.1038/s41467-019-08696-y (PMC6379439; doi:10.1038/s41467-019-08696-y)
Supplement: Supplementary file 13 — Reporting Summary [file 41467_2019_8696_MOESM13_ESM.pdf]

nature research

Corresponding author(s): Kent Hill and Isabel Roditi

Last updated by author(s): Jan 7, 2019

Reporting Summary

Nature Research wishes to improve the reproducibility of the work that we publish. This form provides structure for consistency and transparency in reporting. For further information on Nature Research policies, see Authors & Referees and the Editorial Policy Checklist.

Statistics

For all statistical analyses, confirm that the following items are present in the figure legend, table legend, main text, or Methods section.

n/a	Confirmed	
☐	☒	The exact sample size (*n*) for each experimental group/condition, given as a discrete number and unit of measurement
☐	☒	A statement on whether measurements were taken from distinct samples or whether the same sample was measured repeatedly
☐	☒	The statistical test(s) used AND whether they are one- or two-sided<br>*Only common tests should be described solely by name; describe more complex techniques in the Methods section.*
☒	☐	A description of all covariates tested
☒	☐	A description of any assumptions or corrections, such as tests of normality and adjustment for multiple comparisons
☐	☒	A full description of the statistical parameters including central tendency (e.g. means) or other basic estimates (e.g. regression coefficient) AND variation (e.g. standard deviation) or associated estimates of uncertainty (e.g. confidence intervals)
☐	☒	For null hypothesis testing, the test statistic (e.g. *F*, *t*, *r*) with confidence intervals, effect sizes, degrees of freedom and *P* value noted<br>*Give P values as exact values whenever suitable.*
☒	☐	For Bayesian analysis, information on the choice of priors and Markov chain Monte Carlo settings
☒	☐	For hierarchical and complex designs, identification of the appropriate level for tests and full reporting of outcomes
☒	☐	Estimates of effect sizes (e.g. Cohen's *d*, Pearson's *r*), indicating how they were calculated

Our web collection on statistics for biologists contains articles on many of the points above.

Software and code

Policy information about availability of computer code

Data collection	For motility analysis, data was collected using Adobe Premiere Elements 9.
Data analysis	Analysis of motility trace data was done using the trypanosome tracking algorithm in MATLAB described in: Shimogawa, M. M. et al. Sci Rep. 2018 Jun 14;8(1):9122. doi: 10.1038/s41598-018-27228-0.<br>Statistical analysis of fly infection data was done using Graphpad-Prism version 7.01. (The statistical test used and P-values are reported in figure legends.)

For manuscripts utilizing custom algorithms or software that are central to the research but not yet described in published literature, software must be made available to editors/reviewers. We strongly encourage code deposition in a community repository (e.g. GitHub). See the Nature Research guidelines for submitting code & software for further information.

Data

Policy information about availability of data

All manuscripts must include a data availability statement. This statement should provide the following information, where applicable:

- Accession codes, unique identifiers, or web links for publicly available datasets
- A list of figures that have associated raw data
- A description of any restrictions on data availability

To Whom it May Concern:

There are no accession numbers or datasets associated with the manuscript.

The authors declare that the data supporting the findings of this study are available within the paper and its supplementary information files. Data are available from the corresponding authors upon request. Raw data for all relevant figures are included as a Source Data file. Figures with raw data are: Fig2a-c, Fig3a-b, Fig4a-c, Fig5a-c, Fig6c-d, Suppl Fig 1, Suppl Fig 2h-i, Suppl Fig 3a-b, Suppl Fig 4a, Suppl Fig 5

There are no restrictions on data availability.

Field-specific reporting

Please select the one below that is the best fit for your research. If you are not sure, read the appropriate sections before making your selection.

☒ Life sciences ☐ Behavioural & social sciences ☐ Ecological, evolutionary & environmental sciences

For a reference copy of the document with all sections, see nature.com/documents/nr-reporting-summary-flat.pdf

Life sciences study design

All studies must disclose on these points even when the disclosure is negative.

Sample size	Number of cells to trace for motility trace analysis is based on the number of cells known to be sufficient to reveal a motility defect in known and published motility mutants.<br>Numbers of flies used is based on the authors' knowledge of numbers sufficient to reveal a fly infection defect in known and published fly infection mutants.
Data exclusions	As reported in Revision 1:<br>In the original submission, motility traces used UV-irradiated parasites and showed similar mean squared displacement for PDEB1-KO and wild type parasites. We found through separate studies that UV-irradiation can alter parasite motility and therefore repeated these experiments for the revised manuscript without using UV-irradiation and in these conditions, the PDEB1-KO parasites showed enhanced mean-squared displacement versus wild type. The UV-irradiated motility data were excluded from the revised manuscript because of the potential for UV to impair motility. This information was stated for the editor and reviewers in the response to reviewers.
Replication	As reported in Revison 1:<br>Multiple biological replicates were used for each experiment, as indicated in methods, supplemental methods and figure legends. Almost all replicates of all experiments gave reproducible results. The two exceptions are:<br>1. For assessing fly midgut infections, 2 experiments showed a statistically significant difference between wild type and mutant, while one experiment did not. All experiments are shown and this is noted in the manuscript and the midgut infection is not the conclusion of the paper. We suspect the variability is because these experiments employ complex biological systems - live flies infected with live parasites - so some variability can be expected between different batches of flies and individual infections, as has been reported by other labs. Crucially however, there is a marked and statistically significant defect in the mutant's versus wild type's ability to infect the proventriculus in all experiments, and this is the key message from these experiments.<br>2. In the original submission, motility trace experiments used UV-irradiated parasites and results showed similar mean squared displacement for PDEB1-KO and wild type parasites. We found through separate studies that UV-irradiation can alter parasite motility and therefore repeated these experiments for the revised manuscript without using UV-irradiation and in these conditions, the PDEB1-KO parasites showed enhanced mean-squared displacement versus wild type. Although UV-irradiation was done on both control and mutant samples, we suspect that the UV-irradiated motility data differed because of the potential for UV to impair motility.
Randomization	For fly infections, flies were allocated randomly into groups that were infected with wild type or mutant parasites.
Blinding	Blinding was not done

Reporting for specific materials, systems and methods

We require information from authors about some types of materials, experimental systems and methods used in many studies. Here, indicate whether each material, system or method listed is relevant to your study. If you are not sure if a list item applies to your research, read the appropriate section before selecting a response.

Materials & experimental systems

n/a	Involved in the study	
☐	☒	Antibodies
☐	☒	Eukaryotic cell lines
☒	☐	Palaeontology
☐	☒	Animals and other organisms
☒	☐	Human research participants
☒	☐	Clinical data

Methods

n/a	Involved in the study	
☒	☐	ChIP-seq
☐	☒	Flow cytometry
☒	☐	MRI-based neuroimaging

Antibodies

Antibodies used	Mouse anti-EP: Anti-Trypanosoma brucei procyclin, Purified, (Clone TBRP1/247) (mouse IgG1), Product code: CLP001AP, Supplier name: Cedarlane

	Rabbit anti GPEET: Polyclonal anti-GPEET antibodies (K1) were raised in rabbits using a synthetic peptide, (GPEET)3C, coupled to KLH (Affiniti Research Products Limited, Nottingham, UK) (Ruepp et al., 1997)
Validation	Ruepp et al., 1997; Vassella et al., 2000; Imhof et al., 2014

Eukaryotic cell lines

Policy information about cell lines

Cell line source(s)	Trypanosoma brucei Lister 427 (Bern stock) procyclic forms
Authentication	Cells can be distinguished from other strains by morphology, expression profile of EP and GPEET and population doubling time.
Mycoplasma contamination	Cells were not tested for mycoplasma contamination
Commonly misidentified lines (See ICLAC register)	no commonly misidentified lines used

Animals and other organisms

Policy information about studies involving animals; ARRIVE guidelines recommended for reporting animal research

Laboratory animals	Tsetse flies: Glossina morsitans morsitans
Wild animals	No wild animals were used.
Field-collected samples	No field-collected samples were used.
Ethics oversight	Ethics oversight was not required for fly experiments.

Note that full information on the approval of the study protocol must also be provided in the manuscript.

Flow Cytometry

Plots

Confirm that:

☒ The axis labels state the marker and fluorochrome used (e.g. CD4-FITC).

☒ The axis scales are clearly visible. Include numbers along axes only for bottom left plot of group (a 'group' is an analysis of identical markers).

☒ All plots are contour plots with outliers or pseudocolor plots.

☒ A numerical value for number of cells or percentage (with statistics) is provided.

Methodology

Sample preparation	dsRed measurement: Cells, expressing a cytosolic dsRed were diluted 1:20 in 1x PBS and directly measured.<br>GPEET measurement: Living Cells were span down for 10 minutes, resuspended in 200 ul cold medium containing the primary antibody (polyclonal anti-GPEET antibody, K1, 1:1000) and incubated at 4°C for 30 minutes. After the incubation with the primary antibody, cells were washed twice with cold medium, then resuspended in 200 ul cold medium containing the secondary antibody (Alexa-green 488, 1:1000) and incubated at 4°C for 30 minutes. After the incubation with the secondary antibody, cells were washed twice with cold medium and resuspended in 1 ml 1x PBS and directly measured.
Instrument	NovoCyte Flow Cytometer, model number: Novocyte 2100YB, ACEA Biosciences, Inc.
Software	NovoExpress v1.2.5, ACEA Biosciences, Inc.
Cell population abundance	No sorting was done
Gating strategy	FCS-H threshold was set to larger than 30000. Gating strategy is included in figure S1 for every cell line measured.

☒ Tick this box to confirm that a figure exemplifying the gating strategy is provided in the Supplementary Information.
